# Gamma-irradiated *Aspergillus* conidia show a growth curve with a reproductive death phase

**DOI:** 10.1093/jrr/rrad081

**Published:** 2023-11-07

**Authors:** Shigetoshi Horikiri, Mami Harada, Ryoko Asada, Tetsuaki Tsuchido, Masakazu Furuta

**Affiliations:** Department of Quantum and Radiation Engineering, Graduate School of Engineering, Osaka Prefecture University , 1-2 Gakuen-cho, Naka-ku, Sakai-shi, Osaka 599-8531, Japan; Research Center of Microorganism Control, Organization for Research Promotion, Osaka Metropolitan University, 1-2 Gakuen-cho, Naka-ku, Sakai-shi, Osaka 599-8531, Japan; Department of Quantum and Radiation Engineering, Graduate School of Engineering, Osaka Prefecture University , 1-2 Gakuen-cho, Naka-ku, Sakai-shi, Osaka 599-8531, Japan; Research Center of Microorganism Control, Organization for Research Promotion, Osaka Metropolitan University, 1-2 Gakuen-cho, Naka-ku, Sakai-shi, Osaka 599-8531, Japan; Department of Quantum and Radiation Engineering, Graduate School of Engineering, Osaka Metropolitan University, 1-2 Gakuen-cho, Naka-ku, Sakai-shi, Osaka 599-8531, Japan; Osaka International Research Center for Infectious Diseases, Osaka Metropolitan University, 1-2 Gakuen-cho, Naka-ku, Sakai-shi, Osaka 599-8531, Japan; Research Center of Microorganism Control, Organization for Research Promotion, Osaka Metropolitan University, 1-2 Gakuen-cho, Naka-ku, Sakai-shi, Osaka 599-8531, Japan; Research Center of Microorganism Control, Organization for Research Promotion, Osaka Metropolitan University, 1-2 Gakuen-cho, Naka-ku, Sakai-shi, Osaka 599-8531, Japan; Department of Quantum and Radiation Engineering, Graduate School of Engineering, Osaka Metropolitan University, 1-2 Gakuen-cho, Naka-ku, Sakai-shi, Osaka 599-8531, Japan; Osaka International Research Center for Infectious Diseases, Osaka Metropolitan University, 1-2 Gakuen-cho, Naka-ku, Sakai-shi, Osaka 599-8531, Japan

**Keywords:** fungi, gamma-irradiation, modified logistic model, reproductive cell death

## Abstract

In this study, we evaluated the effects of gamma irradiation on the germination of *Aspergillus* conidia and mycelial growth using microscopy and predictive microbiological modeling methods. A dose of 0.4 kGy reduced the germination rate by 20% compared to the untreated control, indicating interphase death due to the high radiation dose. The number of colonies formed (5.5%) was lower than the germination rate (69%), suggesting that most colonies died after germination. Microscopic observations revealed that mycelial elongation ceased completely in the middle of the growth phase, indicating reproductive death. The growth curves of irradiated conidia exhibited a delayed change in the growth pattern, and a decrease in slope during the early stages of germination and growth at low densities. A modified logistic model, which is a general purpose growth model that allows for the evaluation of subpopulations, was used to fit the experimental growth curves. Dose-dependent waveform changes may reflect the dynamics of the subpopulations during germination and growth. These methods revealed the occurrence of two cell death populations resulting from gamma irradiation of fungal conidia and contribute to the understanding of irradiation-induced cell death in fungi.

## INTRODUCTION

Ionizing radiation has been utilized in the medical and engineering fields to induce cell death in specific cells, such as cancer cells and harmful microorganisms [1]. It causes DNA damage, leading to various functional impairments and eventually cell death [2, 3]. In mouse fibroblasts, low doses of up to 2 Gy can induce reproductive cell death by causing DNA damage, such as double-strand breaks (DSBs). However, in the multi-hit model theory, at doses that cause sublethal damage, DNA repair occurs during the DNA synthesis and gap phases in the cell cycle, allowing for cell recovery [4].

The radiation sensitivity of cells varies depending on cell type and phase. Filamentous fungi are multicellular organisms that grow and proliferate by extending their mycelia and nuclei through mitotic division in hyphal cells. DNA synthesis is not required during conidial germination, and there is a time lag between mycelial cell growth and division accompanied by nuclear synthesis [5]. Several studies have reported the radiation sensitivity of conidia of filamentous fungi and the irradiation dose that causes cell death [6, 7]. However, how radiation-induced DNA damage leads to cell death during growth remains unclear. The survival of filamentous fungi was evaluated by measuring colony formation. However, because these methods only detect cells that can eventually grow, they do not detect temporary growth, such as reproductive cell death. Therefore, determining the effect of the irradiation dose is difficult.

Mathematical predictive models based on growth curves constructed from changes in bacterial density have been developed and used to control microbial growth [8]. Basic growth models have been developed since ancient times and have been used to predict the growth of microorganisms in food. The modified Gompertz [9] and logistic models [10] based on empirical rules have been used in many countries. Furthermore, in recent years, competition models involving complex microbial populations that reflect the actual environment [11] and those that assume recoverable injured bacteria generated using sterilization treatments have been reported [12]. Thus, the properties of each subgroup can be described. However, many of these models are aimed at prokaryotic organisms such as bacteria.

This study considered the actual changes in morphology observed under a microscope in radiation-exposed fungal conidia and applied predictive microbiological models to the growth curves. This allowed the analysis of cell death caused by radiation exposure in fungal conidia. The aim of this study was to elucidate the morphological and growth curve changes in irradiated fungal conidia using microscopy and predictive microbiological models, respectively. Using these methods, we analyzed cell death caused by irradiation of conidia.

## MATERIALS AND METHODS

### Cultivation of fungal strains and preparation of conidia

*Aspergillus niger* NBRC 6342 strain (National Biological Resource Center, NITE, Japan) was cultivated at 28°C for 7 days on potato dextrose agar (PDA; BD Difco Lab., USA) plates. Conidia were collected from the culture plate and washed with a washing solution of sterile 50 mM phosphate-buffered saline (PBS; pH 7.4; WAKO, Japan) containing 2% (w/v) D-glucose (WAKO, Japan) and 0.05% (w/v) Tween 80 (WAKO, Japan). The suspension was filtered through a sterilized gauze and then passed through a 50-μm nylon mesh filter (Filcon S; Becton Dickinson, USA). Conidia were washed twice with the above washing solution, centrifuged (3000 × *g*, 5 min) and resuspended in sterile 50 mM PBS containing 2% (w/v) D-glucose. The suspension contained ~10^7^ conidia ml^−1^ and was used immediately after preparation.

### Gamma ray irradiation

Gamma-ray irradiation was performed using a ^60^Co radiation source in the irradiation room of the irradiation facility at the Radiation Research Center, Osaka Metropolitan University, Japan. Briefly, 1 ml of the prepared conidial suspension was placed in a glass test tube (*φ*20 mm), plugged with an aluminum cap and irradiated on a test tube rack placed at a fixed distance from the ^60^Co source. The irradiation dose ranged from 0.2 to 0.8 kGy, with a dose rate of ~1.87 kGy/h. The samples were tested within 1 h of irradiation.

### Survival assay

The survival rate of gamma-ray-irradiated conidia was measured in terms of colony-forming units. Irradiated or unirradiated conidia were diluted in PBS and spread onto PDA plates. The plates were incubated for 5 days at 28°C, and the colonies were counted.

### Growth curves analysis

The gamma ray-irradiated conidial suspension (0.02 ml) was mixed with 0.18 ml of potato dextrose broth (PDB; BD Difco Lab., USA). Subsequently, 0.2 ml of the mixture (10^5^ conidia ml^−1^) was inoculated into each well of a 96-well microplate. The initial number of conidia in each well was measured at baseline according to the absorbance, and was set within a range that did not increase significantly with respect to the blank. The microplate was statically cultured at 25°C for 3 days in a microplate reader (Thermo Fisher Scientific KK, USA), during which the optical density at 600 nm (OD_600_) was measured approximately every 10 min.

### Microscopic time-lapse imaging analysis

The germination rate and start time as well as the hyphal length of the gamma ray-irradiated conidia were determined. The treated conidia solution was suspended in PDB, and 3 ml of aliquot was inoculated in a 35 mm *φ* plastic dish. Conidia were observed using a phase-contrast microscope (NIKON, Japan, objective lens ×20) equipped with a stage incubator (Tokai HIT, Japan). Briefly, the culture dish was placed in an incubator (25°C) installed on the stage, and microscopic images were continuously generated during incubation in time-lapse mode using a digital camera (NIKON, Japan) every hour for 3 days. From the acquired images, the mycelial length elongating from each conidiophore was determined using the image-processing software Image-Pro (Roper, Japan). The germination rate was calculated by dividing the number of segments from which the emergence of germination tubes was clearly observed at each time point by the total number of segments (200–300 conidia).

## RESULTS

### Survival rate following gamma irradiation

**Fig. 1:**
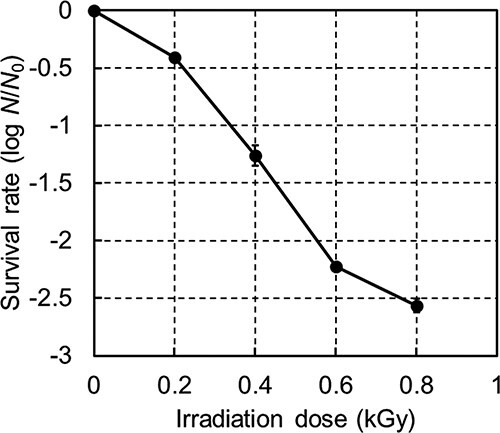
Survival curve of *Aspergillus* conidia in PBS after gamma irradiation (0.2–0.8 kGy), determined using the PDA plate method.

The changes in the colony-forming units of *Aspergillus* treated with gamma irradiation are shown in Fig. 1. The survival rate decreased with higher doses of radiation, being 5.5% (logarithmic value: −1.259 ± 0.088) at 0.4 kGy, and the decimal reduction dose by first-order approximation was 0.28 kGy.

### Time-lapse microscopic observations at the postgermination phase

**Fig. 2:**
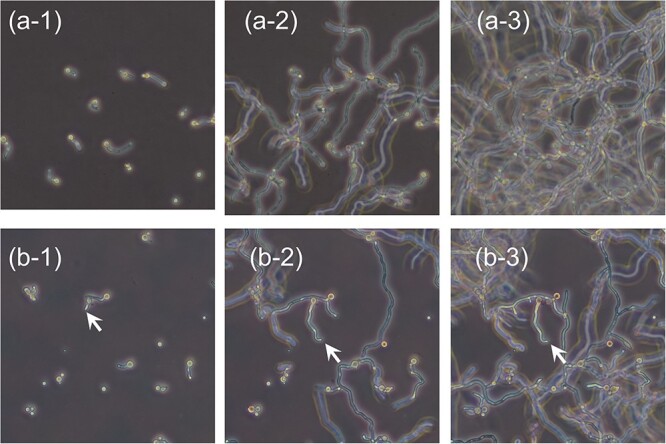
Morphological changes in the conidia and mycelia of 0.4 kGy gamma-irradiated and unirradiated *Aspergillus* grown in PDB. (**a**) Unirradiated, (**b**) gamma-irradiated; 1, 2 and 3 show the state after 12, 18 and 24 h of incubation, respectively.

**Fig. 3:**
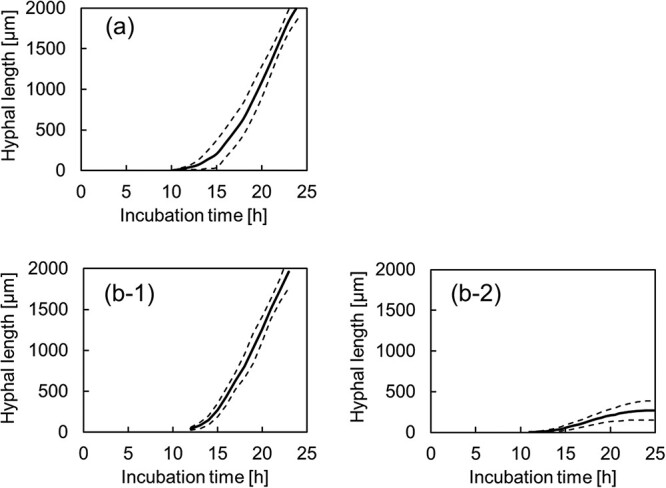
Changes in the mycelial length of 0.4 kGy gamma-irradiated or unirradiated *Aspergillus* after germination in PDB measured using time-lapse microscopy. Mean values of *n* = 5 are shown as solid lines, ± SD are shown as dotted lines. (**a**) Unirradiated and (**b**) 0.4 kGy gamma-irradiated; 1 is typical of a group that showed fast growth and 2 shows a group that stopped growing after germination.

Time-lapse microscopy was performed to observe changes in the mycelia in the early stages of growth after germination (Fig. 2). Unirradiated conidia germinated after 12 h of incubation and exhibited mycelial elongation (Fig. 2a). Similarly, gamma ray-irradiated (0.4 kGy) conidia germinated after 12 h of incubation; however, for conidia indicated with arrows, mycelial growth stopped by 24 h (Figs 2b-2 and 3). Unirradiated (total count: 84) and irradiated conidia (total count: 159) started to germinate at ~8 and 10 h, respectively, and a germination rate of 98.8% was reached at 13 h and that of 69.7% was reached at 18 h, respectively.

The lengths of the mycelia elongating from the conidia were measured (Fig. 3). After germination, unirradiated conidia (Fig. 3a) elongated at a constant rate. On the contrary, 60.0% of irradiated conidia (135 counts out of a total count of 225) elongated as fast as unirradiated conidia (Fig. 3b-1), while 10.7% of irradiated conidia (24 counts) had attenuated mycelial growth (Fig. 3b-2). Irradiated conidia that did not germinate were also observed (29.3%). The length of the mycelium was 260 ± 116 μm (*n* = 5).

The difference (64.2%) between colony-forming rates (5.5%) and germination rates (69.7%) at 0.4 kGy irradiation was due to a slight mycelial elongation after germination, which was attenuated and did not form a colony. This phenomenon is considered to have caused ‘reproductive cell death’, a death in which a cell, starting from a single cell (in this case a conidium) in a quiescent (or interphase) stage, appears to have undergone one or more cell divisions and reproduced (‘proliferated’) as determined using measurements of activity and material content associated with growth, such as turbidity of the culture medium. However, these cells ultimately lose their ability to proliferate during this process without reaching the colony formation stage, and therefore not counted as viable cells using the plate method and are considered to have died.

Conidia are dormant and do not replicate DNA. However, after germination, active cell division occurs and colonies are formed by germination, mycelial elongation and intermycelial fusion. In this study, conidia were irradiated with gamma rays so that mitosis did not occur at the time of irradiation and there was no damage during the division process. In addition, the formation of septum after germination was counted as the first division of conidia, after which they were replaced by mycelia. These spore-specific conditions and the results of time-lapse microscopy showed germination and mycelial elongation (cell division) after irradiation of conidia; the mycelial elongation stopped thereafter. The difference between germination and colonization rates also indicates ‘reproductive cell death’, in which the ability to proliferate is lost.

### Theoretical model for the waveform in growth curves

**Fig. 4:**
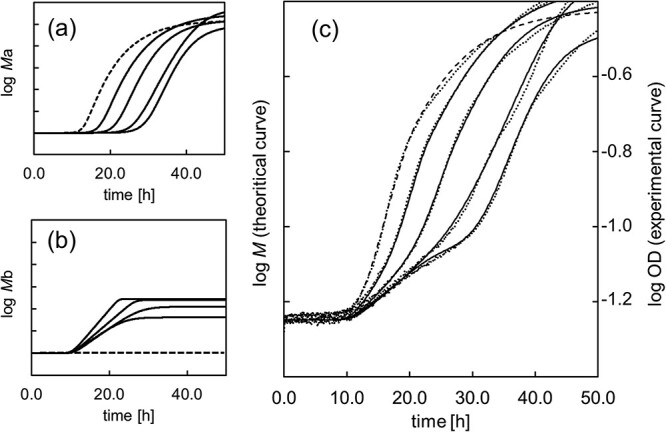
Growth curves showing the change in fungal biomass with time using a modified logistic model. (**a**) Model with increased reference (dashed line) and rise time (*M_a_*); (**b**) model with decreased growth reach and changed specific growth rate (*M_b_*); (**c**) waveform model combining model (a) and model (b) (solid and dashed lines) and experimental growth curve (dotted line). The logarithm of cell density (first axis) represents 1 scale 0.1. The dotted line indicating the experimental values represents the logarithm of the optical density (second axis), together with the level at the start of the culture. In all figures, dashed and solid lines refer to unirradiated and irradiated samples from the short time side.

The proliferation curves of gamma-irradiated conidia based on optical density and a theoretical model are shown in Fig. 4. The growth curves (Fig. 4c, dotted lines) were characterized by distinctive turning points and were considered to reflect the features of the population showing logarithmic proliferation and growth arrest. Therefore, we analyzed the components of each population by fitting a theoretical model to the growth curve. Logistic models, which are widely used as microbial growth models, and the Lotka-Vorttera model [13], which can represent the competitive state of complex microorganisms, have been established. In this study, each component was identified using a modified logistic model [10] that did not include competition coefficients to represent multiple states of the same species (Equation (1)).


(1) \begin{equation*} \frac{dM}{dt}=\mu M\left\{1-{\left(\frac{M}{M_{\mathrm{max}}}\right)}^m\right\}\left\{1-{\left(\frac{M_{\mathrm{min}}}{M}\right)}^n\right\}. \end{equation*}

The vertical axis represents the number of mycelia (*M*), *μ* indicates the specific growth rate, *M*_max_ is the number of mycelium that reached a steady state after growth and *M*_min_ is the number of mycelium at the initial stage of growth. In addition, *m* or *n* indicates a coefficient, where *n* is a parameter involved in the rise time of the logarithmic growth.

Following Tsuchido [14], a model (*M_a_*) was proposed to estimate the number of fungi undergoing logarithmic growth after gamma irradiation. This model was assumed to shift from the waveform of untreated conidia to the long-term side depending on the dose (Fig. 4a) because the growth curve exhibited a delay in the rise time depending on the initial number of fungi. In contrast, another model (*M_b_*) was developed to estimate the number of fungi undergoing growth arrest based on microscopic observations (Fig. 3b-2). These composite waveforms are shown in Equation (2); the curves based on the experimental values and Eq. (2) were fitted using the least-squares method with solver functions (Excel, Microsoft), and the parameters are shown in Table 1.


(2) \begin{equation*} \log M=\log \left({M}_{\mathrm{a}}+{M}_{\mathrm{b}}\right). \end{equation*}

**Table 1:** Gamma radiation dose and each parameter of the corresponding modified logistic model (*M*_*a*_ and *M*_*b*_)

Model	Dose (kGy)	*μ*	*m*	*n*	log *M*_max_
(a) *M*_a_	0.0	3.19	0.15	4.44	8.06
	0.2	2.03	0.21	3.58	8.10
	0.4	1.06	0.55	3.05	8.04
	0.6	0.57	1.40	3.49	8.15
	0.8	0.95	0.82	2.16	7.99
(a) *M*_b_	0.0				
	0.2	0.31	23.4	14.5	7.49
	0.4	0.23	18.0	18.9	7.48
	0.6	0.19	10.0	22.2	7.42
	0.8	0.18	8.2	23.8	7.32

*μ* = specific growth rate, *m* and *n*: variables, log *M*_min_ = cell density in the initial stage, log *M*_max_ = cell density at growth reach. No irradiation in *M_b_* is identical to no irradiation in *M_a_* and is, therefore, not mentioned. The logarithm of cell density indicates the relative value of volume based on the initial cell density (conidia ml^−1^).

**Fig. 5:**
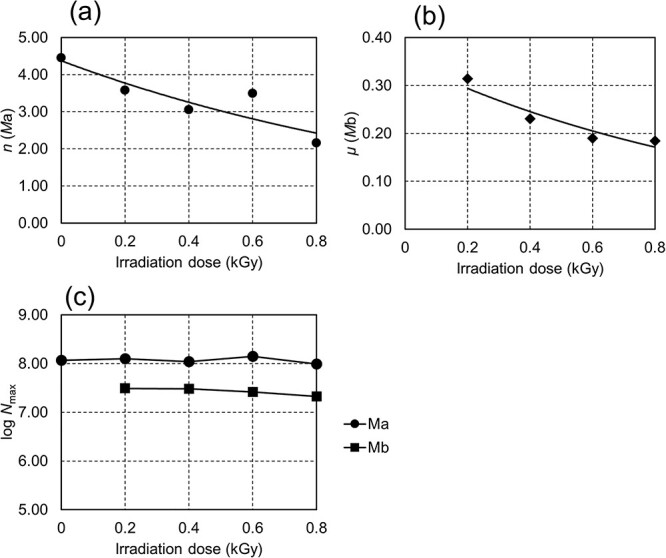
Correlation of each variable in a theoretical model of the growth curve of *Aspergillus niger* with gamma-ray dose (0.2–0.8 kGy). The variable *n* relates to the rise time of the growth curve, *μ* is the specific growth rate, and *N*_max_ is the cell density at growth reach. *M_a_* and *M_b_* indicate each subpopulation of the modified logistic model.

The results of fitting the curves based on Equation (2) to the experimental values are shown in Fig. 4c. The measured curve (dotted line) closely matched the calculated model curve (solid line) in terms of the rise time, slope of the conidia growth curve for each irradiation dose and shape of the inflection point observed at approximately log OD = −1.0 of the measured values (see Supplementary data). When the number of initial cells (*N*_min_) for both *M_a_* and *M_b_* was regarded as variables, the calculation failed to converge. Therefore, *N*_min_ was treated as the constant. In this study, a log*N*_min_ value of 7.0 was used, which is estimated to be sufficiently above the detection sensitivity threshold based on the method employed. *M_b_* assumed a constant carrying capacity because those that grew to a certain cell size were unable to divide and died, which primarily affected the low-concentration curve. The dose-dependent effects on cell numbers for each model can be determined by the parameter *n* in *M_a_*, which relates to the delay time, and by the maximally cell number *N*_max_ in *M_b_*. For viable cells capable of growth without lethal damage, the cell count correlates with the delay time [14]. Furthermore, when cells subjected to lethal damage temporarily increase and then stop proliferating, the maximum cell number (*N*_max_) varies according to the cell count. The relationships between each variable and dose are shown in Fig. 5. In *M_a_*, the parameter *n* demonstrated a decreasing trend with increasing dose (Fig. 5a). The inverse relationship between *n* and *dM*/*dt* suggests a delayed, dose-dependent proliferation, indicating a decrease in the initial cell count. On the contrary, *N*_max_ in *M_b_* showed a gradual decrease relative to *M_a_* and did not exhibit dose-dependence (Fig. 5c). This suggests that *M_b_* reflects characteristics of the preirradiation cell population rather than it being dependent on the extent of damage. Moreover, the specific growth rate *μ* in *M_b_* decreased with increasing dose (Fig. 5b), indicating a potential influence on the proliferation rate.

## DISCUSSION

Exposure of *Aspergillus* conidia to high-dose gamma rays (0.2–0.8 kGy) resulted in several processes leading to cell death. Exposure to radiation, such as X-rays, causes growth arrest in human cells at low doses (<2 Gy). The dose required to reduce the survival rate to 0.1 in a single exposure test against HepG2 cells, which are human-derived cancer cells, is ~6 Gy, except in cases of radiation resistance [15]. Because fungi are known to be resistant to radiation [16], they show a certain proportion of reproductive cell death at doses ranging from 0.2 to 0.8 kGy.

The conidium of *Aspergillus* harbors the genes necessary for growth after germination. Genes coding for DNA repair are expressed during growth [17]. Subpopulations of cells (*M_b_*) that exhibited growth arrest upon gamma irradiation were believed to have undergone lethal DNA damage. It was suggested to be a case of ‘reproductive death’. Our results suggest that the length of mycelia that grew for a short time after germination, within a few rounds of mitosis, falls within a certain range. Filamentous fungi, especially *Aspergillus*, are multinucleate cells and undergo mitosis with the increase in cell size [18]. Mycelial tip cells, which have a high specific growth rate, contain a large number of nuclei [19]. Dormant spores are believed to undergo one to two mitotic divisions during the initial process of germination to form a germ tube, followed by the next mitotic division when the cell size exceeds a critical limit with the elongation of hypha [20]. Our results suggest that mitosis associated with hyphal elongation was discontinued, leading to elongation arrest. This is considered to be a critical factor determining the number of nuclei during the early stage of germination.

In our study, for untreated controls, *Aspergillus* did not form branches up to ~200 μm and then formed branches every 68 μm on an average (data not shown). The average length of the mycelia that stopped growing due to gamma irradiation was ~269 μm, which is assumed to have been attained before branch formation. The formation of branches requires a novel group of proteins, Spitzenkörper, that forms the branching point, and an increase in protein synthesis during mitosis may be necessary for their production [21].

As shown in Fig. 4, *Aspergillus* conidia exposed to gamma radiation were divided into at least two subpopulations during germination and growth by fitting the theoretical formula to the growth curve. The subpopulation (*M_b_*) leading to cell death showed a significantly lower hyphal growth rate than the fast-growing subpopulation (Fig. 3), and the growth rate (*μ*) decreased depending on the radiation dose (Table 1). Multiple subpopulations can exist in a cell population subjected to sterilization treatment, depending on cell injury [22, 23]. Particularly during the elongation process, metabolic processes, such as energy production, substrate production and transport and protein synthesis, are activated, and a decrease in function due to DNA damage can occur over a wide range. Moreover, the growth rate depends on the transport of substances by vesicles necessary for hyphal growth from the hyphal tip and on the production of these substances. Therefore, a decrease in the growth rate reflects these changes in the cells (Fig. 5b). Meanwhile, as shown in Fig. 5c, the Mb subpopulation did not exhibit a dose-dependent increase. This suggests that the number of subpopulations leading to growth inhibition is determined by precharacterized cellular processes, such as the cell cycle.

In the repair of DSBs induced by gamma irradiation, *Aspergillus* is known to preferentially utilize the nonhomologous end joining (NHEJ) pathway involving Ku80, similar to other eukaryotic cells [24]. NHEJ is prone to induce errors and is believed to result in knockouts due to the insertion or deletion of short DNA sequences during DSB repair [25]. In addition, alternative repair pathways distinct from NHEJ, such as microhomology-mediated end joining and single-strand annealing, have also been elucidated; however, both pathways are believed to be error-prone [26]. In our study, the *M_b_* subpopulation, which experienced growth arrest, suggests that either DNA damage was not repaired or normal proliferation was hindered by the accumulation of errors in repair through NHEJ.

On the contrary, the *M_a_* subpopulation significantly differed from the *M_b_* subpopulation that underwent growth arrest, as indicated by the length of hyphae postproliferation. The *M_a_* subpopulation exhibited extensive cell proliferation, far beyond the number of mitotic events observed in the growth-arrested *M_b_* subpopulation, at the same dose. Assuming differences in repair responses depending on the extent of DNA damage, the ratio between the *M_a_* and *M_b_* subpopulations should vary in a dose-dependent manner. However, in reality, the *M_b_* subpopulation did not show an increase with dose. Although the exact cause could not be identified in this study, the findings suggest the presence of at least two distinct subpopulations with different responses to DNA damage and repair processes.

One possible hypothesis for this cause is that a portion of the spore population had already progressed to the S phase. Spores of *Aspergillus nidulans* are believed to get arrested in the G_1_ phase with a single nucleus [27]. However, the spores used in this study were newly formed and immature, raising uncertainty about the uniformity of individual cell characteristics such as the cell cycle. Therefore, some spores are suspected to have transitioned from the G1 phase to the S phase before irradiation. Differences in cell cycle stages may lead to variations in the repair mechanisms postirradiation. After the S phase, sister chromatids are already synthesized and DNA repair through error-free homologous recombination (HR) could take place. Although our results do not solely support this hypothesis, they indicate the existence of distinct subpopulations with different characteristics at the time of irradiation, probably following different pathways even after irradiation.

The delay in the increase of OD is known to be dependent on cell number [22]. We observed that the change in ‘*n*’ (i.e. growth delay) in the *M_a_* subpopulation was likely associated with the change in colony-forming units (Fig. 1). Moreover, the dose-dependent decrease in cell viability indicates that some cells did not exhibit germination or transient elongation.

## CONCLUSION

Our findings revealed temporary proliferation of microorganisms in the gamma-irradiated samples, suggesting that lethal damage may trigger this phenomenon. However, the specific effect of this temporary growth on the samples remains unclear and warrants further investigation. Moreover, potentially lethal damage in reproductively dead cells suggests the possibility of repair and subsequent regrowth under certain conditions. Considering the risk of sublethally injured microbial contamination during sterilization processes [28], the regrowth potential of cells experiencing reproductive death should be assessed to accurately evaluate the risk of microbial recontamination.

## Supplementary Material

Supplementarydata_rrad081

## References

[1] Calado T, Venâncio A, Abrunhosa L. Irradiation for mold and mycotoxin control: a review. Compr Rev Food Sci Food Saf 2014;13:1049–61.

[2] Kuwahara Y, Tomita K, Urushihara Y et al. Association between radiation-induced cell death and clinically relevant radioresistance. Histochem Cell Biol 2018;150:649–59. 30232589 10.1007/s00418-018-1728-z

[3] Jiao Y, Cao F, Liu H. Radiation-induced cell death and its mechanisms. Health Phys 2022;123:376–86. 36069830 10.1097/HP.0000000000001601 PMC9512240

[4] Kelland LR, Steel GG. Inhibition of recovery from damage induced by ionizing radiation in mammalian cells. Radiother Oncol 1988;13:285–99. 3064192 10.1016/0167-8140(88)90224-1

[5] Osherov N, May GS. The molecular mechanisms of conidial germination. FEMS Microbiol Lett 2001;199:153–60. 11377860 10.1111/j.1574-6968.2001.tb10667.x

[6] Goldman GH, McGuire SL, Harris SD. The DNA damage response in filamentous fungi. Fungal Genet Biol 2002;35:183–95. 11929209 10.1006/fgbi.2002.1344

[7] McNamara NP, Black HIJ, Beresford NA et al. Effects of acute gamma irradiation on chemical, physical and biological properties of soils. Appl Soil Ecol 2003;24:117–32.

[8] McMeekin TA, Olley JN, Ross T et al. Predictive Microbiology: Theory and Application. Taunton, UK: Research Studies Press, 1993.

[9] Gibson AM, Bratchell N, Roberts TA. The effect of sodium chloride and temperature on the rate and extent of growth of *Clostridium botulinum* type A in pasteurized pork slurry. J Appl Bacteriol 1987;62:479–90. 3305458 10.1111/j.1365-2672.1987.tb02680.x

[10] Fujikawa H, Kai A, Morozumi S. A new logistic model for bacterial growth. Shokuhin Eiseigaku Zasshi 2003;44:155–60. 12968470 10.3358/shokueishi.44.155

[11] Fujikawa H, Munakata K, Sakha MZ. Development of a competition model for microbial growth in mixed culture. Biocontrol Sci 2014;19:61–71. 24975409 10.4265/bio.19.61

[12] Asada R, Sakamoto JJ, Furuta M, Tsuchido T. Theoretical base for the application of the DiVSaL method to bacterial spores to evaluate injured populations occurring after exposure to lethal stress. Biocontrol Sci 2022;27:169–77. 36216569 10.4265/bio.27.169

[13] Vandermeer JH, Goldber GDE. Population Ecology: First Principles. Princeton, NJ: Princeton University Press, 2003.

[14] Tsuchido T . A novel double subculture method and its theory for the enumeration of injured cells in stressed microbial population. Biocontrol Sci 2017;22:131–5. 28659556 10.4265/bio.22.131

[15] Kuwahara Y, Mori M, Oikawa T et al. The modified high-density survival assay is the useful tool to predict the effectiveness of fractionated radiation exposure. J Radiat Res 2010;51:297–302. 20410675 10.1269/jrr.09094

[16] Saleh YG, Mayo MS, Ahearn DG. Resistance of some common fungi to gamma irradiation. Appl Environ Microbiol 1988;54:2134–5. 3178216 10.1128/aem.54.8.2134-2135.1988 PMC202816

[17] Goldman GH, Kafer E. *Aspergillus nidulans* as a model system to characterize the DNA damage response in eukaryotes. Fungal Genet Biol 2004;41:428–42. 14998526 10.1016/j.fgb.2003.12.001

[18] Trinci APJ, Fiddy C. Mitosis, septation, branching and duplication cycle in *Aspergillus nidulans*. J Gen Microbiol 1976;97:169–84. 796408 10.1099/00221287-97-2-169

[19] Müller C, Spohr AB, Nielsen J. Role of substrate concentration in mitosis and hyphal extension of *Aspergillus*. Biotechnol Bioeng 2000;67:390–7. 10620754 10.1002/(sici)1097-0290(20000220)67:4<390::aid-bit2>3.0.co;2-o

[20] Dörter I, Momany M. Fungal cell cycle: a unicellular versus multicellular comparison. Microbiol Spectrum 2016;4. 10.1128/microbiolspec.FUNK-0025-2016. 28087934

[21] Steinberg G, Peñalva MA, Riquelme M et al. Cell biology of hyphal growth. Microbiol Spectr 2017;5. 10.1128/microbiolspec.FUNK-0034-2016. PMC11687463 28429675

[22] Takano M, Tsuchido T. Availability of growth delay analysis for the evaluation of total injury in stressed bacterial population. J Ferment Technol 1982;60:189–98.

[23] Horikiri S, Furuta M, Tsuchido T. A modified double subculture method for the two-mode injuries evaluation in a stressed fungal spore population. Biocontrol Sci 2020;25:131–8. 32938842 10.4265/bio.25.131

[24] Zhang J, Mao Z, Xue W et al. Ku80 gene is related to non-homologous end-joining and genome stability in *Aspergillus niger*. Curr Microbiol 2011;62:1342–6. 21225265 10.1007/s00284-010-9853-5

[25] Abdulrachman D, Champreda V, Eurwilaichitr L et al. Efficient multiplex CRISPR/Cpf1 (Cas12a) genome editing system in *Aspergillus aculeatus* TBRC 277. J Biotechnol 2022;355:53–64. 35788357 10.1016/j.jbiotec.2022.06.011

[26] Huang J, Cook DE. The contribution of DNA repair pathways to genome editing and evolution in filamentous pathogens. FEMS Microbiol Rev 2022;46:fuac035. 35810003 10.1093/femsre/fuac035 PMC9779921

[27] Bergen L, Morris NR. Kinetics of the nuclear division cycle of *Aspergillus nidulans*. J Bacteriol 1983;156:155–60. 6352675 10.1128/jb.156.1.155-160.1983 PMC215064

[28] Tsuchido T . Double subculture method. In: Tsuchido T and Furuta M (eds). Detection and Control of Spores and Injured Microorganisms in Food Manufacturing and Inspection. Tokyo: CMC Publishing Co., Ltd., 2020, 209–15.

