# Supplementary material for: Gamma-irradiated Aspergillus conidia show a growth curve with a reproductive death phase
Source: J Radiat Res. 2023 Nov 7;65(1):28–35. doi: 10.1093/jrr/rrad081 (PMC10803171; doi:10.1093/jrr/rrad081)
Supplement: Supplementarydata_rrad081 [file supplementarydata_rrad081.docx]

Supplementary data

| Dose (kGy) | *a* | *r* | *p* |
| --- | --- | --- | --- |
| 0.0 | 1.01 | 0.997 | <0.01 |
| 0.2 | 1.10 | 0.997 | <0.01 |
| 0.4 | 1.12 | 0.998 | <0.01 |
| 0.6 | 1.07 | 0.995 | <0.01 |
| 0.8 | 0.98 | 0.997 | <0.01 |
